# Elderly Caregivers’ Awareness of Caregiving Health Risks

**DOI:** 10.3390/healthcare10061034

**Published:** 2022-06-02

**Authors:** Shimon Amar, Aya Biderman, Sara Carmel, Yaacov G. Bachner

**Affiliations:** 1 Department of Family Medicine, Faculty of Health Sciences, Ben-Gurion University of the Negev, Beer-Sheva 84105, Israel; abid@bgu.ac.il; 2 Clalit Health Services, Southern District, Beer-Sheva 84105, Israel; 3 Department of Epidemiology, Biostatistics and Community Health Sciences, Faculty of Health Sciences, Ben-Gurion University of the Negev, Beer-Sheva 84105, Israel; sara@bgu.ac.il (S.C.); bachner@bgu.ac.il (Y.G.B.); 4 Center for Multidisciplinary Research in Aging, Faculty of Health Sciences, Ben-Gurion University of the Negev, Beer-Sheva 84105, Israel

**Keywords:** caregiving burden, awareness of caregiving health risks, primary caregivers

## Abstract

The aim of this study was to assess the level of awareness of elderly primary caregivers of being at physical and mental health risk due to their caregiving role, as well as to examine the impact of sociodemographic characteristics, patient care characteristics, and situational variables on caregivers’ awareness. Data were collected by interview of a sample of primary caregivers aged 60+. A total of 202 primary caregivers responded positively, representing a response rate of 65% (202/311). We found a low–moderate level of awareness. The final multivariate regression analysis (F (12, 179) = 21.26, *p* < 0.000) revealed six variables, out of nearly 30, that are associated with a high percentage (59%) of the variability of caregivers’ awareness, namely caregiving burden, caregivers’ self-rated health, patient’s disease severity, caregiver gender, number of children, and familial relation to the patient. Action may be taken to raise caregivers’ awareness. Such interventions would possibly contribute to the quality of life and health of caregivers, enable the optimal treatment of the patient, and reduce the costs imposed on the health system and society in general.

## 1. Introduction

The worldwide increase in life expectancy has also led to a sharp increase in the number of disabled people [1,2]. In Israel, about 16% of the elderly population living in the community are limited in their daily activities [3,4], and they need the daily assistance and treatment which is provided mainly by family members [5,6]. Most elderly people prefer to be treated at home [7]. Moreover, due to shorter hospitalizations and more outpatient and community care [8], responsibility for these patients is transferred to the patients’ family members. In most cases, care is mainly imposed on one family member, the primary caregiver, most often on the spouses of the elderly who are elderly themselves [9].

Family caregivers may be assisted by the National Insurance Institute through the Long Care Insurance Law, which provides mainly financial support and hired caretakers. Social support is also offered to caregivers by independent nonprofit organizations, such as for Alzheimer’s disease and Parkinson’s disease [10].

The majority of primary caregivers in the community are women [11]. Many of them have significant physical or mental disabilities, while others, such as the adult children of care recipients, are at crucial stages in their private or professional lives and lack the time, tools, or skills required to care for the disabled family member [11].

Caregivers are expected to perform complex and varied tasks daily. It is customary to distinguish between instrumental care tasks, such as shopping, transportation, and arrangements outside the home, and personal care tasks, such as bathing, dressing, or feeding. Not infrequently, those unskilled caregivers are also required to assist in the provision of medications and medical procedures. Occasionally, caregivers are also required to address issues of end-of-life care, considering the growing trends of various home hospice programs and many elderly patients’ desire to end their life at home [8].

Many studies suggest that caregivers in general and older caregivers in particular have become a high-risk group for physical and mental health morbidity and even mortality [12,13]. Psychological symptoms found included depression, anxiety, and emotional stress [14,15], these being most common among caregivers who are the patient’s spouses [16,17], especially among caregivers of elderly patients [18,19]. Physical consequences found included general fatigue [20], digestive and eating problems, reduced immune system activity, slower recovery from injuries [21,22], relatively high levels of blood pressure [23], and many sleep problems [24]. As a result, these caregivers are referred to in the literature as the “hidden victims” [25], with an explicit directive from the World Health Organization to support them and ensure their wellbeing during caregiving and after their relative’s death [26].

The demanding treatment tasks, which require an investment of time, material resources, and physical and mental energy, often lead to a feeling of burden among caregivers [27]. There is a distinction between subjective and objective burden [28]. Objective burden refers to the treatment of the elderly patient, including economic and social difficulties and changes in the social/family role structure, whereas subjective burden reflects the caregivers’ response to the therapeutic situation, such as feelings of shame, insecurity, resentment, and social isolation that accompany treatment [8]. Both feelings of burden have negative consequences for the caregivers’ physical and mental health and for their economic and social wellbeing. In fact, caregiving burden has a negative impact on all areas of the caregivers’ life [29].

Moreover, informal caregivers are less likely to seek help and are, therefore, more vulnerable than any other group to the negative consequences of caregiving. These effects were found to be more severe for caregivers who are elderly themselves [30,31]. Hence, several recommendations agree that general practitioners should actively identify patients who are primary caregivers, assess their health condition on a regular basis, support and treat them, and refer them to the appropriate agencies for assistance [11,32]. Patient care characteristics, situational variables, and levels of awareness were found to have an impact on caregivers’ health according to a literature review and former studies [33,34].

The main goals of this study were twofold: to assess the level of awareness of elderly primary caregivers of being at physical and mental health risk due to their caregiving role, and to examine the associations with caregivers’ sociodemographic characteristics, patient care characteristics and situational variables.

## 2. Methods

### 2.1. Study Population

Data were collected from a sample of primary caregivers that were defined as the person who devoted the most hours per week for caregiving [35]. Inclusion criteria included being a family member aged 60+ and caring for patients suffering from serious diseases such as dementia and cancer. The potential caregivers were recruited from associations for the treatment of severely ill patients, support groups for caregivers, and professional conferences held throughout the country. Eligible caregivers were contacted and given a brief explanation of the study objectives and asked to take part. It was emphasized that there was no obligation to participate, that participation could be stopped at any time, that the gathered information would be used for research purposes only, and that participation was anonymous. Those who agreed underwent a 50 min interview on average, conducted at a designated location of their choice by experienced interviewers who were trained for their role by the study researchers. The data were collected before the COVID-19 pandemic erupted. The study was approved by the ethics committee of Ben-Gurion University of the Negev (approval #21-2014))).

### 2.2. The Study Instruments

We assessed three domains found to have an impact on caregivers’ health according to an in-depth literature review: sociodemographic characteristics, patient care characteristics, and situational variables. For each index, participants were asked to rate their degree of consent on a Likert scale ranging from 1 (not at all) to 4 (very much) or from 1 to 5. The questionnaires included the following: caregiver awareness of health risk due to caregiving (four items) derived from the Health Belief Model [36], α = 0.92. A low score in each index indicates a low level of awareness. The degree of involvement in patient care activities (seven items) was measured using an index developed by Bachner [8], α = 0.83. Caregiving burden perception (12 items) was assessed using the abridged Zarit Burden Interview (ZBI) [37], α = 0.81. For social support (two items) and the perception of the physician’s interest in the caregiver (six items), α = 0.72, we developed a dedicated questionnaires for this study following an extensive literature review. The questionnaires were reviewed by two healthcare professionals (a physician and a medical sociologist), experts in the field of informal caregiving, to ascertain face validity. Self-efficacy (10 items) was measured using the index developed by Sherer et al. [38], α = 0.89. Caregivers’ perception of their health status was measured using two items (*r* = 0.87) [39].

Sociodemographic characteristics of the caregivers included age, gender, marital status (married/cohabiting/single), number of children, country of birth (Israel/other), degree of religiosity (secular/traditional/religious/ultrareligious), level of education (nonacademic/academic), financial status (bad or mediocre/good or very good), and employment status (employee/nonemployee).

Patient care characteristics included family relation to the patient (spouse/children/other), duration of treatment, severity of patient illness (mild/moderate/severe/very severe), living arrangements (living with the patient or not), length of care (months, days, hours), assistance in caring for the patient (assisting others/not assisting others), caring for other relatives (yes/no), past caregiving experience of family members with either non-sick or serious illnesses (yes/no), in-home foreign worker (yes/no), and caregiving burden.

Situational variables included involvement in patient care activities, family doctor’s interest in the caregiver’s health, self-efficacy, social support, number of visits to a family doctor, and caregivers’ self-rated health.

### 2.3. Statistical Analysis Methods

Descriptive statistics were used to describe the data (means, standard deviation, and percentages). Associations between the dependent and independent variables were examined using Pearson, Spearman, or chi-square tests according to the scale’s structures. The internal consistency of the study indices was examined using Cronbach’s alpha. The unique contribution of the independent variables to the explanation of the dependent variables was examined using a multi-hierarchical linear regression analysis. Only variables that were found to be statistically significant in the bivariate analyses were included in the regression equations. SPSS software v. 21 (IBM, Armonk, NY, USA) was used for data processing and analysis. Statistical significance was set at *p* < 0.05.

## 3. Results

A total of 311 primary caregivers were offered the chance to participate in the study, 202 of whom responded positively, representing a response rate of 65% (202/311). Table 1 shows that the caregivers’ mean age was 70.73 years (SD 10.52), and 68.2% were women, of whom 57.9% were spouses of the ill family member. Most were married (88%), with a mean of 3.1 children (SD 1.4), and over one-third (38.9%) had an academic education. The vast majority were retired from work (65.2%), while 34.8% worked outside their home. Most caregivers (58.5%) defined themselves as being in a good or very good financial condition. The care recipients’ profiles, as obtained by the caregivers’ reports, indicated that their mean age was 77.51 years (SD = 15.69), and about half were men (51.5%). Most had an elementary or high-school education (60.4%), and the vast majority did not work (91.1%). Care recipients suffered from dementia (32.2%), cancer (28.9%), and other physical diseases (38.9%; Cerebro Vascular Accident (CVA), Parkinson’s disease, heart diseases, Chronic Obstructive Pulmonary Disease (COPD), and rheumatic diseases).

As for the caregiving variables, Table 1 shows that the caregivers provided prolonged care to an ill family member, with an average rate of 86.77 (SD 148.76) months, 5.84 (SD 2.12) days a week, and 14.75 (SD 9.63) h per day on average. More than one-third (37.1%) of caregivers reported that the patient’s illness was severe. Most caregivers (65.2%) lived with the patient, and 60.2% were assisted by additional family members in the patient’s care. More than half of caregivers reported not having previously cared for other family members who were seriously ill (54.8%). Moreover, 38.8% reported that they were assisted by a foreign worker, and 62.6% of caregivers reported that they were not participating in a support group.

The level of caregivers’ awareness of the treatment harm was found to be low–medium (M = 2.64, SD = 1.25) relative to the scale range (1–5). For instance, for the item “Does caring for a family member cause significant harm to your health?”, 23.3% responded that the treatment did not harm their health at all, while only a minority (10.9%) said that caregiving caused harm to their health to a very large extent.

Associations between all independent variables and the level of awareness of treatment risks were first examined using bivariate analyses according to a variable scale structure (Table 2).

All independent variables found to have a significant association with awareness level were entered into three multiple regression analysis models according to the domains of the variables (Table 3).

Regarding sociodemographic characteristics, five variables were found to have a significant unique contribution (F (7, 187) = 6.53, *p* <0.000) to explaining the level of awareness. In order of intensity of their contribution, these were number of children, degree of religiosity, economic status, sex, and country of birth. These variables explained a relatively moderate percentage (19%) of the variability of awareness, whereas both education and employment status had no significant contribution to the awareness of treatment risks. Regarding the caregiving characteristics, three variables were found to have a significant unique contribution (F (6, 181) = 12.74, *p* < 0.000) to explaining the level of awareness. In order of intensity of their contribution, these were number of treatment days per week, degree of patient’s disease severity, and primary caregiver being the spouse of the patient. These variables explained a moderate percentage (29%) of the variability of awareness. Residence with the patient and hours of treatment per day were found to be not significant. Among the situational indices, four variables were found to have a significant unique contribution (F (7, 192) = 25.87, *p* < 0.000) to explaining the level of awareness. In order of intensity of their contribution, these were caregiving burden, self-perception of health status, involvement in treatment activities, and primary care physician’s interest in the patient. These variables explained a relatively high percentage (47%) of the variability of the dependent variable. Self-efficacy, social support, and number of visits to the family physician were not found to have a significant unique contribution to explaining the level of caregivers’ awareness.

The final multivariate regression analysis included the variables found to have a significant contribution to the explanation of caregivers’ awareness of health risks in the three former regressions (Table 4).

Results of this analysis (F (12, 179) = 21.26, *p* < 0.000) revealed six variables with a significant unique contribution. According to their intensity, they were caregiving burden, caregivers’ self-rated health, disease severity, caregiver sex, number of children, and familial relation to the patient. These variables explained a very high percentage (59%) of the variability of caregivers’ awareness. Therefore, the level of caregivers’ awareness of their health risk was related to higher caregiving burden, higher perception of severity of the patient’s disease, worse perception of health status, being a woman, and having fewer children. Furthermore, caregivers who were spouses of the patient had a higher level of awareness compared to caregivers who were the patient’s adult children.

## 4. Discussion

Considering caregiving implications, this study found that caregivers continue to be mostly unaware of the negative impact of the care they provide to ill relatives. Hence, it is highly important to understand the factors that are related to their awareness so that intervention programs can be developed. Thus, the second aim of our study focused on associations between caregivers’ awareness and their sociodemographic characteristics, patient care characteristics, and situational variables.

In the final multivariate regression, six variables were significantly associated with caregivers’ awareness of health risks due to caregiving: caregiving burden, caregiver’s self-rated health, severity of the patient’s illness, caregiver’s gender, number of children, and familial relation to the patient.

Caregiving burden and caregiver’s self-rated health were the strongest predictors of caregivers’ awareness of treatment risks. Caregivers who perceived a lower burden and a better health status were less aware of their risks. The association between these variables was high, since both expressed the totality of the relationship with the caregiver’s suitability to the treatment needs. These two variables were also shown to be highly important for caregivers’ wellbeing by Abdollahpour et al. [40]. However, since these two variables are subjectively reported, high variance across caregivers should be assumed [41]. Both variables are influenced by caregivers’ personal, cultural, and behavioral characteristics; therefore, they do not necessarily reflect the objective burden or health status. Despite our preference for a subjective report measure, representing the caregivers’ point of view, it should be noted that other integrative modalities for measuring burden and wellbeing have been offered [42]. The same is true for the severity of the patient’s illness, which was also subjectively reported and significantly associated with higher awareness of health risks. Hence, health professionals are advised to routinely assess caregivers’ burden and actively address the issue of their health risks [11]. According to our findings, this is particularly important for caregivers who do not feel burdened, who self-rate their health as better, and whose patient’s disease is perceived as less severe.

The remaining three significant predictors of awareness of health risks, which were all caregivers’ personal nonmodifiable characteristics, revealed that male caregivers, those who had more children, and those who were not the spouse of the patient had a lower level of awareness of care-derived health risks.

This gender difference might be related to the differences in caregiving activities between women and men. Whereas women are reported to provide personal physical care in day-to-day activities, emotional support, housekeeping, and out-of-home arrangements, men are primarily engaged in care-related arrangements and tend to seek help from others [8]. Such differences may affect the sense of physical and emotional burden of the treatment and, hence, the awareness of its risks. These findings are in accordance with other studies [43] which found that spousal care is more burdensome for women than it is for men. On the other hand, another study [44] found that caregivers’ burden fluctuates over time. This suggests that it is necessary to develop interventions that take into consideration the particularities of the care situation [45].

The finding that a higher number of children is associated with lower awareness may be due to the caregivers’ experience of support and a more balanced distribution of caregiving burden among all family members. This is in accordance with the finding that nearly two-thirds of caregivers reported that they receive familial help with caregiving duties. We found the spouse’s level of awareness to be significantly higher than that of other family caregivers. This finding was described in previous studies [46], particularly in reference to their account of providing longer and more meaningful care to their spouses.

As a whole, we suggest two different explanations for the relatively low–moderate reported awareness of all caregivers. First, at the level of public awareness, low awareness may be due to limited public attitude toward and interest in the issue from both the health system and the media. Therefore, the individual caregiver does not perceive their role as significant and does not consider the side-effects of the treatment itself [47]. Second, in terms of family structure and functional models, we should be aware of features unique to each family, as well as cultural values and behaviors that influence the perception of the role of each member of the household, particularly the spouse [48]. These are expressed by feelings of mutual guarantee, marital commitment beyond marriage, personal responsibility, and more, leading caregivers to ignore either the burden of care intensity or the impact on their health. Nevertheless, caregivers report that such a burden exists inherently.

Other treatment variables, which were expected to be significantly associated with caregiving awareness, as noted in Cochrane et al.’s systematic review [49], were found to be statistically significant in the bivariate analysis but not in the multivariate analysis. These include the degree of religiosity and financial status, along with the number of treatment days per week and involvement in patient care activities. We can assume that, here, there is also a case under-reporting and low self-awareness which stems from the patterns we described earlier. On the one hand, it is reasonable that these characteristics affect the caregivers’ burden; on the other hand, these characteristics are pushed aside when caregivers feel obligated toward their duty, preventing further considerations from disturbing their caregiving task.

According to our findings, we suggest that medical and welfare agencies should allocate direct resources to support family caregivers, such as community-level interventions, to address needs for physical and mental assistance. Medical professionals need support and guidance to encourage and develop their competencies to assess and support family caregivers through medical follow-ups and preventative measures [50]. Enrichment programs for physicians should be part of the specialization program in family medicine and other professions that deal with the care of the elderly in the community [51,52]. It is also advisable to develop measurement tools for evaluating the impact of interventions provided by physicians who have undergone such training on the physical and emotional state of caregivers. Intervention programs for physicians and for caregivers should complement each other to empower caregivers and their resources. Such interventions would possibly have an impact on the quality of life and health status of caregivers and the optimal treatment of the patient, in addition to delaying institutionalization and potentially reducing the costs imposed on the health system and society in general.

### Limitations of the Study

The present study was based on a single interview of caregivers (cross-sectional) and on a convenience sample. This means that causality could not be inferred and that the sample does not represent the entire population of elderly caregivers. Moreover, selection bias was possible, as those who agreed to participate in the study might have been more aware of the issue in the first place or had higher self-confidence. It is also possible that events that occurred shortly before the interview affected the interviewees’ reports and could not be filtered. In addition, the use of questionnaires might have triggered a social desirability bias in the caregivers’ responses to some of the questions.

## 5. Conclusions

The number of primary caregivers for the elderly in the community is expected to increase in the coming years. Although the risks of caregiving are well known, our findings demonstrate that caregivers have a relatively low–moderate level of awareness of these risks. Caregivers who feel less burdened, who perceive a better self-rated health, and whose patient’s disease is perceived as less severe have lower awareness. Caregivers who are not the patient’s spouses are also less aware of the risks, thus necessitating special attention. Raising caregivers’ awareness of their health risks is important due to its potential impact on their preventative health behaviors, their lower tendency to seek social and medical help, and their ability to provide optimal care to the patient. Further studies related to caregivers’ awareness of their health risks in other countries and in different healthcare systems are needed to expand and support our findings.

## Figures and Tables

**Table 1:** Descriptive statistics of caregivers’ sociodemographic characteristics, patient care characteristics, and situational variables (*n* = 202).

Variable	Mean (SD) Range
Age (years)	70.73 (8.33)
	60–89
Number of children	3.11 (1.44)
	0–13
Total length of care (months)	86.77 (14.87)
	1–96
Number of days of care (Per week)	5.84 (2.12)
	5–7
Number of hours of care (Per day)	14.75 (9.63)
	5–24
Identity of the patient family member	***n*** **(%)**
Spouse	117(57.9)
Son/daughter	29 (14.4)
Another family member	56 (27.7)
Residence	
With the patient	70(34.8)
Without the patient	131 (65.2)
Gender	
Males	64 (31.8)
Females	137 (68.2)
Marital status	
Married/cohabits	176 (88)
Other	24 (12)
Country of birth	
Israel	89 (44.3)
Other	112 (55.7)
Degree of religiosity	
Secular	124(62.3)
Traditional/Religious	75 (37.7)
Level of education	
Academic	77 (38.9)
Nonacademic	121 (61.1)
Employment status	
Employed	70 (34.8)
Unemployed (retired)	131 (65.2)
Assistance in caregiving	
None	80 (39.8)
Any	121 (60.2)
Severity of patient illness	
Mild	6 (3)
Moderate	56 (27.7)
Severe	75 (37.1)
Very severe	65 (32.2)
Caring for other patients	
Yes	31 (15.4)
No	170 (84.6)
Treatment of non-sick family members	
Yes	45 (22.3)
No	157 (77.7)
Past care for seriously ill family members	
Yes	84 (41.6)
No	118 (54.8)
In home foreign worker	
Yes	59 (38.8)
No	93 (61.2)
Support group	
Yes	49 (37.4)
No	82 (62.6)

**Table 2:** Associations between all independent variables in three domains (sociodemographic characteristics, patient care characteristics, and situational variables) and level of awareness of health risks, using bivariate analyses according to variable scale structure (*n* = 202).

Variable	No. of Items	Range	Mean (SD)	Association with Awareness of Health Risks
Sociodemographic characteristics				
Age			70.73 (8.33)	*r* = 0.11 NS
Number of children			3.11 (1.44)	*r* = −0.16 *p* < 0.05
Gender				
Male			2.31 (1.21)	*t* = 2.66
Female			2.81 (1.23)	*p* = 0.08
Marital status				
Unmarried			2.46 (1.46)	*t* = −0.80
Married			2.68 (1.22)	NS
Country of birth				
Israel			2.36 (1.25)	*t* = −3.05
Other			2.89 (1.20)	*p* = 0.003
Degree of religiosity				
Secular			2.46 (1.24)	*t* = −2.84
Traditional/religious			2.97 (1.21)	*p* = 0.005
Education				
Nonacademic			2.80 (1.25)	*t* = 2.16
Academic			2.41 (1.22)	*p* = 0.03
Employment status				
Unemployed **(retired)**			2.80 (1.25)	*t* = 2.16
Employed			2.41 (1.22)	*p* = 0.03
Financial status				
Poor/medium			2.96 (1.12)	*t* = 3.13
Good/very good			2.43 (1.29)	*p* = 0.002
Patient care characteristics				
Residence with the patient				
No			2.07 (1.12)	*t* = −5.06
Yes			2.96 (1.21)	*p* = 0.00
Family support				
No			2.59 (1.29)	*t* = −0.56
Yes			2.69 (1.21)	NS
Caring for another sick family member				
No			2.68 (1.27)	*t* = 1.15
Yes			2.40 (1.11)	NS
Treatment of an additional patient				
No			2.72 (1.27)	*t* = 1.54
Yes			2.39 (1.14)	NS
Past care for a sick family member				
No			2.67 (1.23)	*t* = 0.35
Yes			2.61 (1.27)	NS
In home foreign worker				
No			2.59 (1.22)	*t* = 0.17
Yes			2.56 (1.18)	NS
Support Group				
No			2.41 (1.28)	*t* = −1.55
Yes			2.75 (1.09)	NS
Total length of care (months)				*r* = 0.01 NS
Number of days of care per week				*r* = 0.41 *p* < 0.001
Number of hours of care per day				*r* = 0.35 *p* < 0.001
Family relation to the patient				F(b,w) = 9.91 (2, 199) *p* < 0.001
Spouse			2.9 (1.17)	
Son/daughter			2.76 (1.14)	
Another family member			2.04 (1.13)	
Severity of patient illness				F(b,w) = 10.85 (3, 198) *p* < 0.001
Mild			1.58 (1.31)	
Moderate			2.15 (1.09)	
Severe			2.57 (1.10)	
Very severe			3.25 (1.28)	
Caregiving burden	11	1–4.45	2.39 (0.79)	*r* = 0.57 *p* < 0.001
Situational variables				
Involvement in patient care activities	7	1–5	3.15 (1.06)	*r* = 0.34 *p* < 0.001
Doctor’s interest in the caregiver	6	1–4	1.42 (0.61)	*r* = 0.22 *p* < 0.001
Self-efficacy	10	1.4–4	3.02 (0.65)	*r* = −0.24 *p* < 0.001
Social support	2	1–5	3.57 (1.08)	*r* = −0.19 *p* < 0.01
Number of visits to a family doctor				*r* = 0.19 *p* < 0.01
Caregivers self-rated health	2	1.5–6	3.78 (0.85)	*r* = −0.44 *p* < 0.001

**Table 3:** Results of a multiple linear regression analysis to explain the level of caregivers’ awareness of health risks by sociodemographic characteristics, caregiving characteristics, and situational variables (*n* = 202).

**Awareness of health risks by sociodemographic characteristics**				
Variable	B	S.E	β	t
Number of children	−0.20	0.06	−0.23	−3.22 **
Degree of religiosity +	0.57	0.19	0.22	3.07 **
Financial status ++	−0.47	0.17	−0.18	−2.70 **
Gender &	0.4	0.18	0.15	2.22 *
Country of Birth &&	0.35	0.17	0.14	2.05 *
Education ^	−0.16	0.18	−0.06	−0.91
Employment status ^^	−0.11	0.18	−0.04	−0.61
***R***^2^ = 0.19, * ***p*** < 0.05, ** ***p*** < 0.01,				
**Awareness of health risks by patient care characteristics**				
Caregiving burden	0.65	0.09	0.41	7.11 ***
Number of treatment days per week	0.17	0.05	0.29	3.40 ***
Severity of patient illness #	0.39	0.09	0.26	4.06 ***
Number of treatment hours per day	0.02	0.01	0.18	1.86
Caregiver is a spouse of the patient ##	0.57	0.28	0.15	2.01 *
Caregiver is a son/daughter of the patient	0.12	0.24	0.05	0.51
Residence with the patient @	0.08−	0.28	−0.03	−0.27
***R***^2^ = 0.29, * ***p*** < 0.05, *** ***p*** < 0.001				
**Awareness of health risks by situational variables**				
Caregivers’ self-rated health	−0.40	0.08	−0.27	−4.52 ***
Involvement in patient care activities	0.19	0.07	0.16	2.66 **
Family physician’s interest in caregiver health	0.25	0.11	0.12	2.20 *
Self-efficacy	−0.15	0.12	−0.08	−1.30
Social support	0.07	0.06	−0.06	−1.11
Number of visits to a family doctor	0.01	0.02	0	0.04
***R***^2^ = 0.47, * ***p*** < 0.05, ** ***p*** < 0.01, *** ***p*** < 0.001				

+ 1—secular, 2—traditional/religious; ++ 1—medium economic condition, 2—good economic condition; & 1—male, 2—female; && 1—Israel, 2—other; ^ 0—nonacademic, 1—academic; ^^ 0—does not work or retired, 1—works; # 1—mild, 2—modest, 3—severe, 4—very severe; ## 1—yes, 0—no; @ 1—no, 2—yes.

**Table 4:** Results of final linear regression analysis to explain the level of caregivers’ awareness of health risks (*n* = 202).

Variable	B	S.E	β	t
Caregiving burden	0.69	0.08	0.44	8.05 ***
Caregivers’ self-rated health	−0.42	0.08	−0.28	−5.28 ***
Severity of patient illness #	0.36	0.07	0.24	4.66 ***
Gender &	0.43	0.13	0.16	3.34 ***
Number of children	−0.11	0.05	−0.13	−2.27 *
Caregiver is a spouse of the patient ##	0.43	0.18	0.12	2.38 *
Number of treatment days per week	0.05	0.03	0.09	1.55
Family physician’s interest in caregiver’s health	0.12	0.11	0.06	1.13
Degree of religiosity +	0.13	0.14	0.05	0.89
Financial status ++	0.11	0.14	0.04	0.84
Country of birth &&	0.07	0.13	0.03	0.59
Involvement in patient care activities	0.02	0.07	0.01	0.27
***R*^2^** = **0.59**, * *p* < **0.05**, *** *p* < **0.001**				

+ 1—secular, 2—traditional/religious; ++ 1—medium economic condition, 2—good economic condition; & 1—male, 2—female; && 1—Israel, 2—other; # 1—mild, 2—modest, 3—severe, 4—very severe; ## 1—yes, 0—no; The continuous variables ranged from low to high.

## Data Availability

The principal investigators had full access to all the data in the study and take responsibility for the integrity and accuracy of their analysis. The data presented in this study are available on request from the corresponding author.
